# Monitoring resistance of *Plasmdium vivax*: Point mutations in dihydrofolate reductase gene in isolates from Central China

**DOI:** 10.1186/1756-3305-4-80

**Published:** 2011-05-17

**Authors:** Fang Huang, Shuisen Zhou, Shaosen Zhang, Weidong Li, Hongwei Zhang

**Affiliations:** 1 National Institute of Parasitic Diseases, Chinese Center for Disease Control and Prevention; WHO Collaborating Centre for Malaria, Schistosomiasis and Filariasia; Laboratory of Parasite and Vector Biology, Ministry of Health, Shanghai 200025, PR China; 2 Department of Parasitology, Anhui Center for Disease Control and Prevention, Hefei 230061, PR China; 3 Department of Parasitology, Henan Center for Disease Control and Prevention, Zhengzhou 450003, PR China

## Abstract

**Background:**

Malaria still represents a significant public health problem in China, and the cases dramatically increased in Central China after 2001. Antifolate resistance in *Plasmodium vivax *is caused by point mutations in genes encoding dihydrofolate reductase (*pvdhfr*) and dihydropteroate synthase (*pvdhps*). In this study, we used direct sequencing to investigate genetic variation in *pvdhfr *of malaria patients' samples from Central China.

**Results:**

Among all the samples, 21.4% were wild-type, whereas mutations were detected at three codons (58, 61 and 117) including single mutant (34.6%) and double mutants (43.8%). The most prevalent mutant allele was the one with double mutation at codons 58 and 117 (24.6%). Three types of single mutation (S58R, T61M and S117N) were found in 2.1%, 11.8% and 20.9% of parasite isolates, respectively. The four *P. vivax *parasite populations in Central China also differed in *pvdhfr *allele frequencies.

**Conclusions:**

This study suggested that *P. vivax *in Central China may be relatively susceptible to pyrimethamine. And it also highlights genotyping in the *pvdhfr *genes remains a useful tool to monitor the emergence and spread of *P. vivax *pyrimethamine resistance.

## Background

*Plasmodium vivax *is the major cause of malaria outside Africa, mainly in Asia and Americas [1]. Although responsible for less mortality than *P. falciparum*, *P. vivax *causes considerable morbidity, particularly in children [2]. In China a total of 14491 malaria cases and 59741 suspected cases with 12 deaths were reported by the annual case reporting system in 2009 and more than 90% of the total cases were vivax malaria cases [3]. In Central China, the re-emergence of malaria was considerable in provinces along the Huang-Huai River, especially in Anhui and Henan Provinces. The malaria prevalence increased considerably with the highest incidence and over half of the total malaria cases in 2006 in Anhui Province [4].

Since it is difficult to monitor the susceptibility of *P. vivax *to antimalarial drugs by *in vitro *tests [5], molecular markers of drug resistance are useful tools for mapping the current and changing pattern of pyrimethamine resistance of *P. vivax *isolates. Antifolate resistance is strongly associated with mutations at specific sites in the gene encoding *dhfr *in malaria parasites [6]. In protozoans, *dhfr *and thymidylate synthases are parts of a bifunctional enzyme encoded by a single gene. Some research showed that by *in vitro *assays and kinetic studies of recombinant DHFR enzymes there are several codons that may undergo amino acid substitutions [7-9].

To date, a number of non-synonymous mutations including codons 33, 57, 58, 61, 117 and 173 in the DHFR domain of *P. vivax*, have been reported worldwide [10-17]. These mutant *dhfr *encode amino acid sequences which are similar to those that cause antifolate resistance in *P. falciparum*, which suggests that antifolate resistance in *P. vivax *could have arisen in the same manner as that found in *P. falciparum*. It was also very likely that antifolate-resistant *P. vivax *was selected as a result of heavy deployment of antifolates against co-existing falciparum malaria and other infections. While this work was in progress, the kinetic properties of both wild-type and mutant (S58R+S117N) recombinant *pvdhfr *were reported [9]. Mutations in *pvdhfr *of codons 58 and 117 are considered to be equivalent to *pfdhfr *mutations at residues 59 and 108, respectively, which are associated with pyrimethamine.

In China, an action plan for malaria elimination was proposed by the Ministry of Health last year. The predominant malaria parasite *P. vivax *in Central China has demonstrated resilience to elimination and become increasingly prevalent in Central China for the past 10 years [18]. With vivax malaria outbreaks occurring frequently in many counties of central provinces in recent years, vivax malaria has become predominant parasite species and is responsible for more than 90% of malaria cases in China. According to the antimalarial drug policy of China, the first-line therapies for vivax malaria treatment are chloroquine and primaquine, which have been used for more than 60 years, and there is increasing evidence showing the emergence and spread of chloroquine resistance in vivax, especially in Southeast Asia [19].

Most of the studies on antifolate resistance in vivax were performed in regions where vivax and falciparum coexist. Because vivax malaria has not been directly treated with antifolate drugs, the evolution of mutation in the *pvdhfr *gene could only be inferred from possible selection by treatment of falciparum malaria with antifolate drugs. Until now, mutations in *pvdhfr *has been studied from limited vivax samples from temperate zone countries, where vivax is the predominant malaria parasite species and has a dramatically different relapse phenotype compared with tropical strains [20]. In the present study, *P. vivax *isolates were collected from four different sites in Central China, and the sequence of the entire *dhfr *domain was determined to investigate genetic variation in *P. vivax *dihydrofolate reductase.

## Methods

### Study area

**Figure 1:**
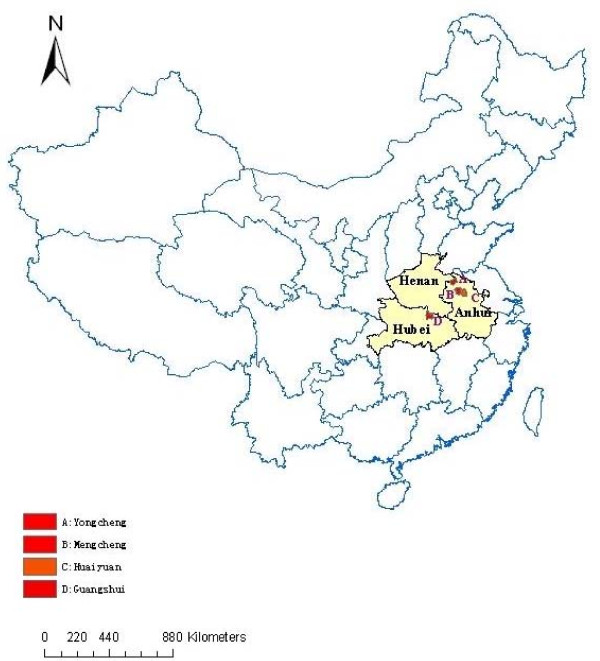
**Map of distribution sentinel sites and mutant *pvdhfr *in Central China**.

In recent years the re-emergence of malaria was considerable in Central China, especially along the Huang-Huai River. Four counties in three provinces located in Central China were selected for collecting blood samples (Figure 1). The counties were Huaiyuan and Mengcheng county in Anhui Province, Yongcheng county in Henan Province, Guangshui city in Hubei Province. All the counties were located at 32°17'~34°18' north latitude, 113°~117°09' east longitude with malaria re-emergence in recent years.

### Sample collection

A total of 187 blood samples from patients with acute *P. vivax *infections with signs and symptoms associated with malaria were collected from various clinics/hospitals in different geographical locations in Central China.

The diagnosis was made by microscopic examination of Giemsa-stained thin and thick blood smears, and then a multiplex PCR was performed to confirm the *Plasmodium *species. All the patients were found to be infected only with *P. vivax*. An additional drop of blood from finger pricked capillary blood was collected on 3M Whatman filter papers for storage and transport of infected blood samples after informed consent was obtained. The filter papers were thoroughly dried, sealed in a plastic bag with desiccant, and stored at -20°C.

All the blood samples were collected with the consent of each patient and approval of the protocol was obtained from the Institutional Research Ethical Committee prior to the study. Patients with a positive smear were treated with chloroquine (total of 25 mg/kg body weight in three divided daily doses) plus primaquine (total of 180 mg in eight divided daily doses) according to national guideline for treatment of *P. vivax *infections.

### Parasite DNA extraction and amplification

The filter papers were thoroughly rinsed in 500 μl of sterile distilled water, placed in micro tube to which 50 μl of distilled water were added. DNA was extracted by Chelex method [21].

A multiplex PCR using conditions described previously [22] was performed to confirm the Plasmodium species in all isolates.

Based on the complete *dhfr-ts *sequence (GenBank accession nos.98123), including 5-upstream non-coding region, the following primer pairs were designed for the nested PCR protocol: The primary amplification was performed using the external primers, VDT-OF (5'-ATGGAGGACCTTTCAGATGTATTTGACATT-3') and VDT-OR (5'-GGCGGCCATCTCCATGGTTATTTTAT CGTG-3'), wherein the entire *P. vivax dhfr-ts *gene (1.8 kb) was amplified. This primary amplification product was then used for performing nested PCR to amplify *pvdhfr *domain (711 bp). The oligonucleotide pair used was VDT-OF (5'-ATGGAGGACCTTTCAGATGTATTTGACATT-3') and VDFNR (5'-TCACACGGGTAGGCGCCGTTGATCCTCGTG-3').

The reaction mixture consisted of template DNA (10 μl of genomic DNA extracted from filter papers for the primary amplification and 1 μl of the primary reaction for the secondary nested PCR), 15 pmol of forward and reverse primers, buffer (50 mM KCl, 10 mM Tris, pH 8.3), 1.5 mM MgCl_2_, 200 μM deoxynucleoside triphosphates, and 1 unit of *Taq *DNA polymerase (Promega, USA) in a final volume of 50 μl. The PTC-100 thermal cycler (PTC-200, Bio-Rad, USA) was programmed as follows: 94°C for 2 min for the first cycle and 30 s in subsequent cycles, 50°C for 1 min for the first cycle and 30 s in subsequent cycles, and 72°C for 1 min for all cycles, for a total of 30 cycles, followed by a 15 min extension step at 72°C. The amplified PCR products were then analyzed on 2% agarose gel, stained with ethidium bromide, and visualized under ultraviolet illumination. All the samples showed specific amplification in the present study.

### Sequence analysis

The amplified products were purified from agarose gel and sequenced by automated DNA sequence (ABI systems, Perkin-Elmer, France). Sequence alignments and analysis was carried out using Mega and Bioedit software. Amino acid sequences were compared with wild-type sequences (GenBank accession nos.98123 for *pvdhfr*)

## Results

### Malaria situation

The malaria transmission in Central China was very low in 1990's, and malaria was basically eliminated in most areas with malaria incidence less than 1/10,000. However, early in the 21^th ^century, malaria has reemerged in these areas, especially the Anhui Province.

**Figure 2:**
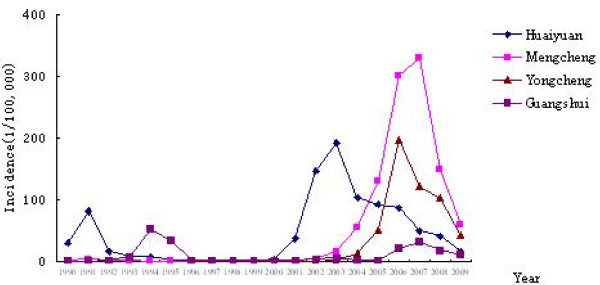
**Malaria incidences in Huaiyuan, Mengcheng, Yongcheng and Guangshui Counties from year 1990 to 2009 from case report system**.

From Figure 2, malaria reemerged in Yongcheng, Mengcheng and Huaiyuan Counties from 2000 and the incidence of Yongcheng County was highest with 122.41/100,000 in 2007 while malaria is relatively stable in Guangshui City. In these areas, there was a plain landscape with soybean as the primary crop, and it was a fixed vectorial area, including *An. sinensis *and *An. anthropophagus *based on historical vectorial investigations in the Huang-Huai River region. The average vector capacity in this area was 0.1686 in 1990's.

Zhou [23] found that the spatial distribution between malaria cases and water-body, the changing of meteorological factors, and increasing vectorial capacity and basic reproductive rate of *An. sinensis *lead to malaria re-emergence in these areas.

### Mutations in the *pvdhfr *gene

**Figure 3:**
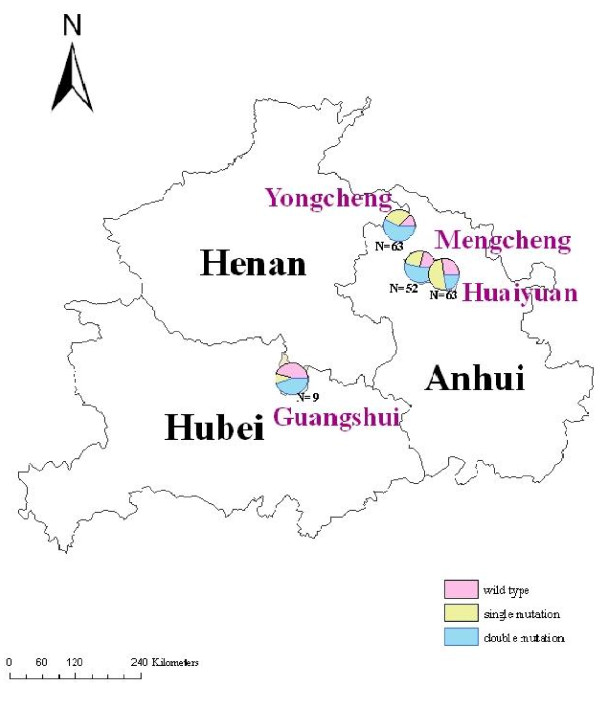
**The Pie charts show the proportions of wild type and mutant *pvdhfr *alleles**.

Total of 187 samples were collected and all the samples were confirmed to be *P. vivax *by PCR (data not shown). *Pvdhfr *gene sequences from a total of 187 *P. vivax *samples were amplified by primary and nested PCR. Sequence polymorphism was assessed in *pvdhfr *genes from 187 *P. vivax *samples. Compared with the wild-type sequence (GenBank accession nos.98123), *pvdhfr *genes from the 187 samples had point mutations, among which three resulted in amino acid substitutions. No synonymous mutations were detected at positions 58, 61 and 117, and the point mutation at codons 57 and 173 was not observed in the samples. The S117T mutation was the most prevalent (20.9%), followed by the T61M mutation. Among all the samples, 21.4% were wild-type, whereas mutations were detected at three codons (58, 61 and 117) including single mutant (34.8%) and double mutants (43.8%) without triple mutations. The proportions of wild type and mutant *pvdhfr *alleles were different in different provinces (showed in Figure 3).

On the basis of amino acid changes, the *pvdhfr *sequences can be grouped into six alleles. Except for the wild-type allele, the most prevalent mutant allele was the one with double mutation at codons 58 and 117 (24.6%). Three types of single mutation (S58R, T61M and S117N) were found in 2.1%, 11.8% and 20.9% of parasite isolates, respectively. The four *P. vivax *parasite populations in Central China also differed in *pvdhfr *allele frequencies (Table 1). 147 of 187 samples yielded identical *dhfr *sequence which was of mutant type. These isolates carried the key double mutations associated with pyrimethamine resistance, S58R and S117N, which correspond to Cys59Arg and Ser108Asn substitutions in *P. falciparum*, respectively.

**Table 1:** Prevalence of *pvdhfr *mutant alleles in *P.vivax *isolates from Central China

Region	*Pvdfhr*					
	
	N(%)	57	58	61	117	173
Wild type		F	S	T	S	I
Huaiyuan County	17(27.0)	F	S	T	S	I
	0	F	R	T	S	I
	9(14.3)	F	S	M	S	I
	23(36.5)	F	S	T	N	I
	10(15.9)	F	R	T	N	I
	4(6.3)	F	S	M	N	I
Mengcheng County	11(21.2)	F	S	T	S	I
	2(3.8)	F	R	T	S	I
	4(7.7)	F	S	M	S	I
	7(13.5)	F	S	T	N	I
	17(32.6)	F	R	T	N	I
	11(21.2)	F	S	M	N	I
Yongcheng County	8(12.7)	F	S	T	S	I
	2(3.2)	F	R	T	S	I
	9(14.3)	F	S	M	S	I
	8(12.7)	F	S	T	N	I
	15(23.8)	F	R	T	N	I
	21(33.3)	F	S	M	N	I
Guangshui City	4(44.4)	F	S	T	S	I
	0	F	R	T	S	I
	0	F	S	M	S	I
	1(11.2)	F	S	T	N	I
	4(44.4)	F	**R**	T	N	I
	0	F	S	M	N	I
Total	187					

These changes and corresponding positions of mutations observed in *pvdhfr *were shown in Table 1. Various numbers of wild-type alleles were identified from parasites originating from different regions with frequencies ranging from 12.7% to 44.4%.

## Discussion

In terms of morbidity, *P. vivax *infections account for a considerable part of malarial disease and economic burden in China and elsewhere in Southeast Asia, calling for increasing research effort to understand the epidemiology of *P. vivax *[2].

The current standard treatment of *P. vivax *for chloroquine-sensitive infections recommended by WHO is chloroquine 25 mg base/kg bw divided over 3 days, combined with primaquine 0.25 mg base/kg bw, taken with food once daily for 14 days to eliminate hypnozoites[24]. In China, the first line drugs for the current treatment of *P. vivax *are chloroquine plus primaquine and the total dosage of primaquine (180 mg dosage for 8 days) is different from WHO recommendation. The second line drugs was artemisinin combined therapy (ACT), which includes four types [25].

Mutations in the *pvdhfr *gene are known to be associated with resistance to antifolate drugs. Although antifolates were used to treat *P. falciparum *malaria, very little information was available about the origin and spread of this drug resistance in *P. vivax*. Allelic diversity at flanking microsatellite loci of the *dhfr *gene will be helpful in understanding the origin and spread of antifolate resistance in *P. vivax *populations as well as the evolutionary history of the parasite species. Therefore, we planned to analyze the extent of these mutations in *P. vivax *populations in Central China, by isolating and sequencing the *dhfr *gene for 187 *P. vivax *isolates originating from different geographical regions of Central China. This is the first study to investigate *pvdhfr *polymorphisms in Central China.

In the present study, we have analyzed *pvdhfr *gene sequences from 187 *P. vivax *isolates from different geographical regions in Central China (Figure 1). Analysis of *pvdhfr *sequences showed limited polymorphism as compared to earlier studies. Approximately half of the *P. vivax *isolates obtained in Central China present the key double Arg-58/Asn-117 mutations in the *dhfr *gene and it is similar to that of the neighboring countries, Cambodia [26], Myanmar [15], Thailand [27] and Vietnam [28]. But we did not find triple and quadruple mutations just as that of recent study in China, Thailand, Vietnam and Korea [29,30]. Likewise, none of the Cambodia, Thailand, and Myanmar isolates studied had the mutant Leu-173, homologous to Ile164Leu substitution in the *P. falciparum dhfr *associated with a high level antifolate resistance. However, in contrast to Thailand isolates, which showed a high proportion (54%) of mutant Leu-57, all isolates in our studies had the wild type Phe-57 codon.

Pyrimethamine was used for radical treatment of *P. vivax *combined with primaquine 40 years ago [unpublished data]. Also, pyrimethamine was added to salt for prophylaxis in 1980's. Pyrimethamine plus primaquine were recommended as prophylaxis of medicine for specific populations in China in 1980's [31]. Surprisingly, in our study there is a difference in *pvdhfr *allele frequencies between the sentinel sites, but the reason was not clear. So far, no data is available on its susceptibility to pyrimethamine. There were different population structures and genetic polymorphisms in *P.vivax *and it could be postulated that they have a different susceptibility to pyrimethamine. Besides, it has been hypothesized that the S117N mutation represents the first step in the drug-resistance selection process that has occurred in the parasite [32].

For vivax malaria resistance in China, only one report showed the data of *in vitro *susceptibility to chloroquine of *P. vivax *isolate in Central China and the geometric mean IC50 of chloroquine was 63.23 nmol/L and although 6 isolates showed resistance to chloroquine *in vitro*, we cannot conclude that there was chloroquine resistance in China [33].

## Limitation

Although our study showed that *P. vivax *in Central China may be relatively susceptible to pyrimethamine, it was not recommended in national antimalarial drug policy for the treatment or prophylaxis of vivax malaria treatment. A previous study [33] has found vivax isolates are resistant to chloroquine *in vitro*, therefore some related to chloroquine-resistance gene will be tested in the future.

## Conclusions

However, from the molecular viewpoint, it is of interest to analyze, as in the case of *P. falciparum*, to what extent a molecular marker, or set of markers, can predict the parasitological and clinical outcome of antimalarial drug treatment. Further molecular characterization of *P. vivax *isolates from different endemic areas may be a useful alternative approach to establish the epidemiology of drug-resistant malaria.

## Conflict of interests

The authors declare that they have no competing interests.

## Authors' contributions

FH and SSZ organized the survey, coordinated and supervised the sample collection, data entry and analysis. LHT helped in the study design and choice of study villages to be included in the trial and reviewed the manuscript. SSZ performed Parasite DNA amplification and the quality controls of all blood samples results. WDL and HWZ processed all the filter papers collection. All authors read and approved the final manuscript.
